# Impact of Asthma on Plantar Pressures in a Sample of Adult Patients: A Case-Control Study

**DOI:** 10.3390/jpm11111157

**Published:** 2021-11-07

**Authors:** Roi Painceira-Villar, Vanesa García-Paz, Ricardo Becerro de Bengoa-Vallejo, Marta Elena Losa-Iglesias, Daniel López-López, João Martiniano, Héctor Pereiro-Buceta, Eva María Martínez-Jiménez, Cesar Calvo-Lobo

**Affiliations:** 1 Research, Health and Podiatry Group, Department of Health Sciences, Faculty of Nursing and Podiatry, Universidade da Coruña, 15403 Ferrol, Spain; roi.painceira.villar@udc.es (R.P.-V.); daniellopez@udc.es (D.L.-L.); hector.pereiro@udc.es (H.P.-B.); vanesa.garcia.paz@sergas.es (V.G.-P.); 2 Departament of Allergology. Complexo Hospitalario Universitario de Ferrol, 15403 Ferrol, Spain; 3 Facultad de Enfermería, Fisioterapia y Podología, Universidad Complutense de Madrid, 28040 Madrid, Spain; ribebeva@ucm.es (R.B.d.B.-V.); cescalvo@ucm.es (C.C.-L.); 4 Faculty of Health Sciences, Universidad Rey Juan Carlos, 28922 Alcorcon, Spain; marta.losa@urjc.es; 5 Escola Superior de Saúde da Cruz Vermelha Portuguesa, 1300-125 Lisbon, Portugal; jmartiniano@esscvp.eu

**Keywords:** asthma, plantar pressure, adults, body posture

## Abstract

Based on the high prevalence of asthma in the population, and its relationship with various musculoskeletal and postural disorders, the aim of this study was to evaluate the plantar pressures in asthmatic patients compared to a control group. A case-control study involving 90 participants was conducted, consisting of 45 asthma patients and 45 healthy paired controls. Static plantar pressure data were recorded using a portable pressure sensor platform. Statistically significant differences were shown in the body weight on the left heel (*p* = 0.031), and the right forefoot maximum peak pressure was lower in the case group (*p* = 0.042). The findings of this study show alterations in static plantar pressures in asthmatic patients compared to healthy individuals. Specifically, the subjects with asthma showed less maximum pressure in the right forefoot and less weight on the left heel, which appear to be associated with the asthma disease.

## 1. Introduction

Asthma is the most prevalent respiratory pathology worldwide, according to World Health Organization data, affecting some 300 million people [1] and being more prevalent in adults of the female sex [2].

Today, it is recognized as a complex and multifactorial condition with an idiopathic etiopathogenesis [3]. In addition to the characteristic lung involvement that is usually evaluated by forced spirometry, this pathology has been associated with musculoskeletal disorders [4], decreased quality of life [5], reduced functional capacity, sedentary behavior [6], and alterations in mechanosensitivity [7], which represent major public health problems.

In addition, the increase in epidemiological studies showed an augmentation of the prevalence of various postural alterations in patients diagnosed with asthma, such as increased dorsal kyphosis and lumbar hyperlordosis [4], altered head and shoulder posture [8], and elevation and anteversion of the shoulder girdle [9].

Furthermore, musculoskeletal alterations and changes in posture in asthmatic patients are usually associated with orthopedic alterations, and are usually the cause of a high demand for regular care visits and consultations with physicians [10]. We hypothesized that asthma may cause a change in the plantar pressures of these patients.

The current literature clearly presents the impact of orthopedic alterations on quality of life, pain, plantar pressures, and functionality. These include hallux valgus [11], Sever's disease [12], deformities in elderly patients [13], and hyperkeratotic lesions [14].

The biomechanics of the locomotor system is based on an interrelation between the different body segments, which is why it is essential to evaluate the impact of these postural changes on the individual's posture as a whole [15].

The analysis of posture and plantar pressures using a pressure platform has been used to assess postural influence on various pathologies, such as diabetic neuropathy [16], rheumatoid arthritis [17], and obesity [18].

Given the importance of the postural impact on asthmatic patients, an analysis of their foot posture and plantar pressures is necessary, in order to determine possible clinical actions that are focused on improving their body posture.

The main objective of our research was to evaluate the plantar pressures in asthmatic patients compared to the healthy general population.

## 2. Material and Methods

### 2.1. Study Design

This study was designed and carried out following the Strengthening the Reporting of Observational Studies in Epidemiology (STROBE) [19] guidelines. This case-control study was used to assess the postural impact and plantar pressures in asthmatic patients and healthy matched controls.

### 2.2. Ethical Statement

This study has a favorable report from the Research Ethics Committee at the Universidade da Coruña (Spain). Before participating in the study, all included subjects signed an informed consent form.

This research complies with all current regulations on human experimentation, as well as the Declaration of Helsinki [20].

### 2.3. Sample Size Calculation

Sample size calculation was performed considering the differences between two groups for independent samples using G * Power software (version 3.1.9.2). This calculation was based on the body weight on the left heel plantar pressure values of a pilot study (*n* = 40) with the following 2 groups (mean ± SD) : 20 asthma patients (25.35 ± 2.27%) and 20 healthy participants (26.95 ± 3.50%). In addition, a 1-tailed hypothesis, an effect size with a Cohen’s d of 0.54, an α error probability of 0.5, and a power of 0.80 were used for the sample size calculation. Considering the previous data, a sample size of 86 participants, 43 cases and 43 healthy matched controls, was calculated.

### 2.4. Sample

The recruitment of the participants was carried out in the allergology area of the Hospital Universitario de Ferrol between February 2019 and November 2019, using a consecutive sampling method; a private clinic was used for the case group and the population of patients with external consultations was in the same hospital as the control group (A, Coruña, Spain). 

In this research, the following inclusion criteria were established: subjects aged between 18 and 65 years; not treated with allergy immunotherapy; active non-smokers or ex-smokers; those who had previously signed the informed consent form. The case group included patients who presented self-reported values related to a positive bronchodilator test showing an FEV1 greater than 200 mL and 12% with respect to the reference values [21]. The control group was made up of healthy matched subjects.

Those subjects under 18 years of age or over 65, those treated with allergy immunotherapy, active smokers or ex-smokers, those who did not sign the informed consent form, and/or people diagnosed with systemic, psychiatric, neuropathic, musculoskeletal pathologies and/or cancer were excluded from the sample.

### 2.5. Sociodemographic and Descriptive Data

Quantitative descriptive data such as age (years), weight (kg), height (m), body mass index (BMI; calculated using the Quetelet index as kg/m^2^) were also collected [22].

### 2.6. Primary Outcome Measures

A portable platform with pressure sensors (Medicapteurs, Balma, France) was used to carry out the measurement of subjects’ static plantar pressures [23]. The S-Plate software for Windows (Medicapteurs, Balma, France) was used to carry out the collection and management of the data obtained. This platform is reliable and reproducible in its measurements, it calibrates itself with each use, and it has been used in numerous investigations [24,25,26].

**Figure 1:**
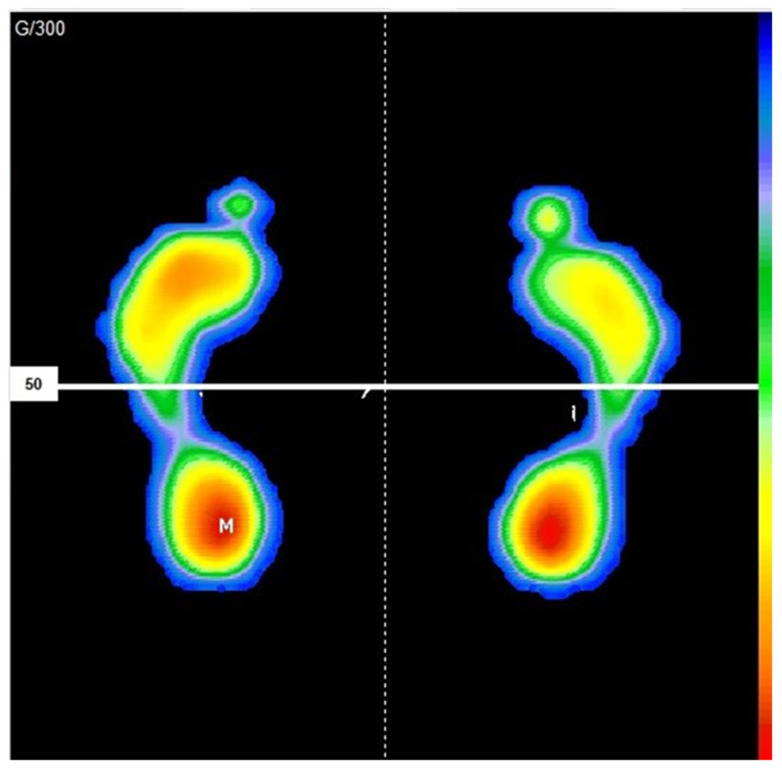
Static analysis of plantar pressures using S-Plate software.

During the assessment of the static plantar pressures, participants were instructed to look straight ahead and keep their arms relaxed at their sides. Six valid 30-second measurements were performed, as previously described, ruling out the tests where the participant moved [12] (Figure 1).

The data collected from these measurements were as follows: total surface area (cm^2^), forefoot surface area (cm^2^), heel surface area (cm^2^), body weight on the lower limbs (%), body weight on the forefoot (%), body weight on the heel (%), average peak pressure (g/cm^2^), forefoot maximum peak pressure (g/cm^2^) and heel maximum peak pressure (g/cm^2^). All these data were obtained using S-Plate plantar pressure analysis software.

### 2.7. Statistics

Statistical procedures were performed using the Statistical Package for Social Sciences (SPSS version 24.0; IBM; Armonk-NY; IBM Corp., New York, NY, USA), considering an α error of 0.05 (*p* value < 0.05 as statistically significant) for a 95% confidence interval (CI).

The Kolmogorov–Smirnov test (using Lilliefors correction) was used to assess the distribution of normality. Parametric data were described as mean ± standard deviation (SD) and range (minimum–maximum values). Parametric data (according to a Kolmogorov–Smirnov test with a value of *p* ≥ 0.05) were compared with Student's *t*-test for independent variables (according to Levene's test). Non-parametric data (according to a Kolmogorov–Smirnov test with a value of *p* < 0.05) were compared using independent samples from the Mann–Whitney U test.

Considering the categorical data, frequencies were used to detail these values. Gender differences between the case and the control groups were determined with Fisher's exact test.

Furthermore, bivariate correlational analyses were carried out in asthma patients and healthy controls in order to correlate outcome measurements, which showed previously statistically significant differences between both groups (percentage of body weight on the left heel and right forefoot maximum peak pressure), with the sociodemographic and descriptive data (age, weight, height, and BMI). Pearson’s (*r*) and Spearman’s (*r*_s_) correlation coefficients were analyzed according to parametric or non-parametric distribution, respectively, and categorized into weak (from 0.00 to 0.39), moderate (from 0.40 to 0.69), strong (from 0.70 to 0.84), and very strong (from 0.85 to 1.00). These analyses were completed with scatter plots for the statistically significant correlations. Finally, multivariate predictive analyses were applied using linear regressions to predict these outcome measurements, which showed previously statistically significant differences between both asthma and control groups, based on the descriptive and sociodemographic data. Linear regression analyses were carried out with the stepwise selection method determining the *R*^2^ coefficient in order to state the quality adjustment. The sociodemographic and descriptive data (sex, age, weight, height, and BMI) were considered as independent variables, while percentage of body weight on the left heel and right forefoot maximum peak pressure were considered as dependent variables [7].

## 3. Results

### 3.1. Sociodemographic and Descriptive Data

Of the total 90 recruited participants, half were patients diagnosed with asthma (case group; *n* = 45) and the other half were healthy matched subjects (control group; *n* = 45). The sample included a total of 31 men and 59 women (%). The ages of the participants in the sample were between 19 and 65 years.

No significant differences were observed between the case group and the control group, in relation to the qualitative sociodemographic and descriptive data (Table 1).

### 3.2. Primary Outcome Measures

The primary outcomes are shown in Table 2. When we evaluated the static plantar pressures, we found that the body weight on the left heel was lower in the case group compared to the control group.

A statistically significant decrease in the right forefoot maximum peak pressure was also detected in the case group, which appears to be associated with the asthma disease.

No statistically significant differences were found between the two groups in the total surface area, left forefoot surface area, left heel surface area, right forefoot surface area, right heel surface area, body weight on the lower left limb, body weight on the lower right limb, body weight on the left forefoot, body weight on the right forefoot, body weight on the right heel, average peak pressure, left forefoot maximum peak pressure, left heel maximum peak pressure, and right heel maximum peak pressure.

### 3.3. Correlational Analyses

**Figure 2:**
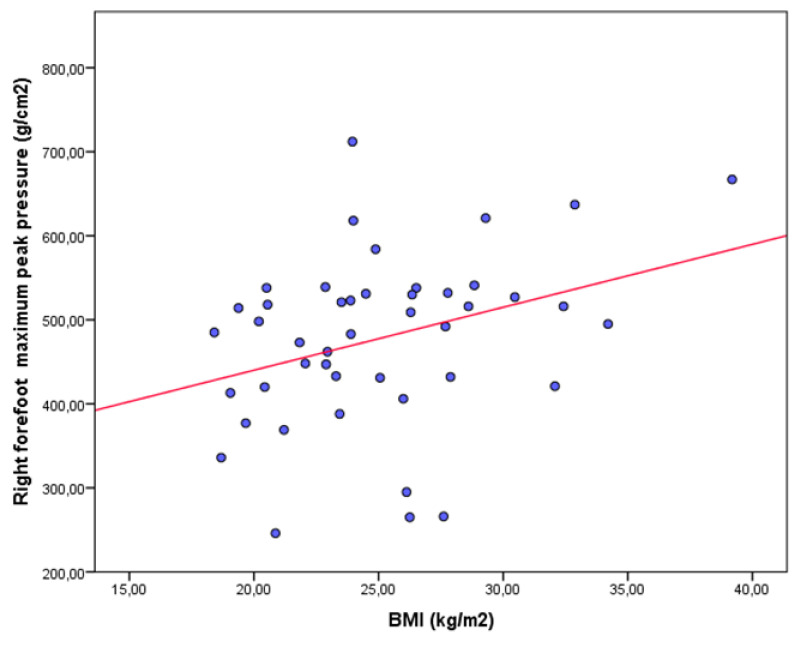
Scatter plot showing a significant positive weak correlation between the right forefoot maximum peak pressure and BMI in patients with asthma.

**Figure 3:**
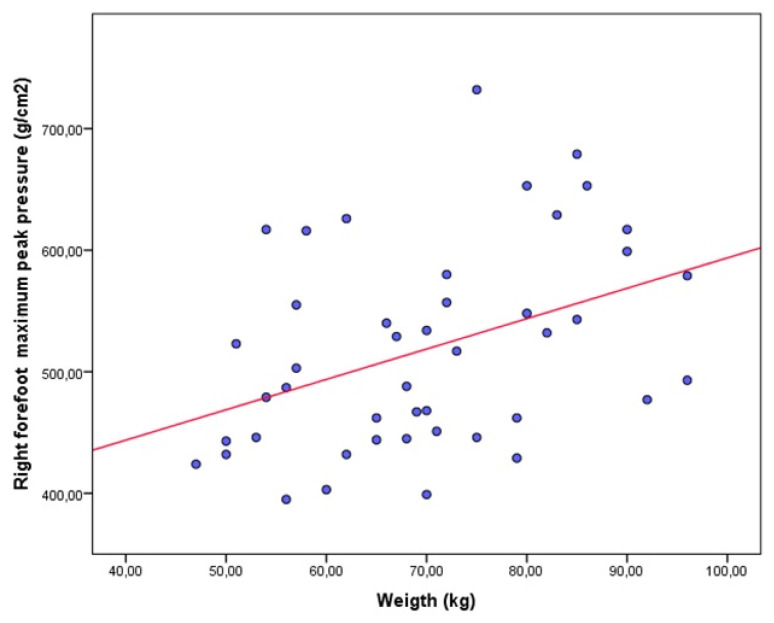
Scatter plot showing a significant positive weak correlation between the right forefoot maximum peak pressure and weight in healthy participants.

**Figure 4:**
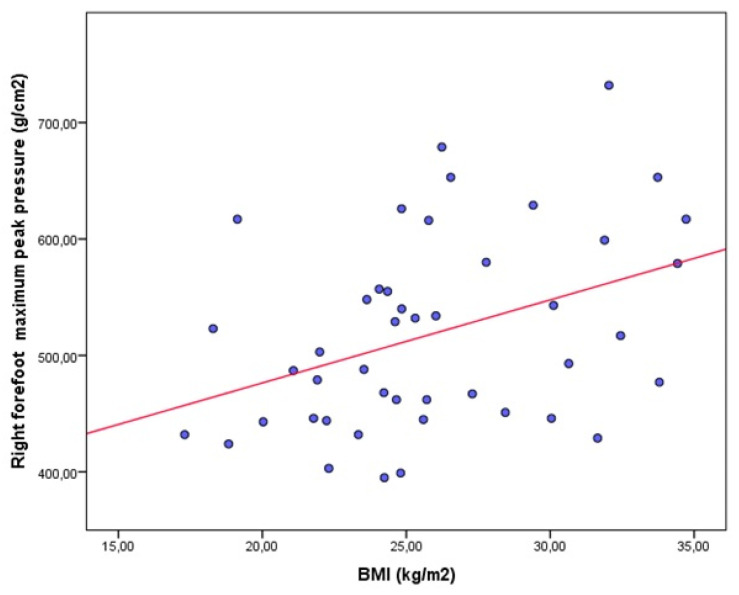
Scatter plot showing a significant positive moderate correlation between the right forefoot maximum peak pressure and BMI in healthy participants.

**Figure 5:**
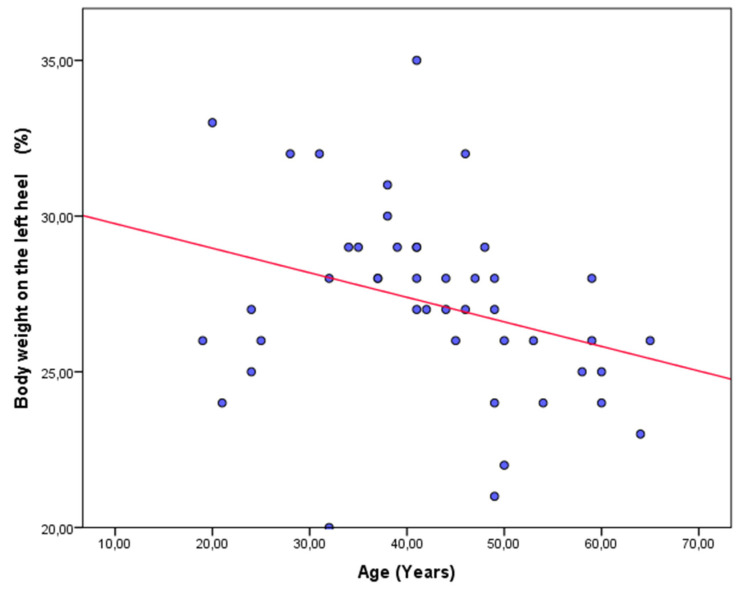
Scatter plot showing a significant negative moderate correlation between the body weight on the left heel and age in healthy participants.

The results from the bivariate correlational analyses are shown in Table 3 for the asthma and control groups. In the asthma group, a statistically significant positive weak correlation was only shown between the right forefoot maximum peak pressure and BMI (Figure 2). In the healthy group, significant positive correlations, from weak to moderate, were shown between the right forefoot maximum peak pressure and weight (Figure 3), and BMI (Figure 4), as well as significant negative correlations, from weak to moderate, between the body weight on the left heel and age (Figure 5).

### 3.4. Multivariate Predictive Analyses

A significant linear regression model (*p* = 0.030; F_1.88_ = 4.874) showed that the body weight on the left heel was only predicted by the study group (*R*^2^ = 0.052; β = −1.444), while a lower body weight on the left heel was determined for the asthma group. Another significant linear regression model (*p* < 0.001; F_1.88_ = 13.655) showed that the right forefoot maximum peak pressure was only predicted by the BMI (*R*^2^ = 0.134; β = +7.662), indicating a higher right forefoot maximum peak pressure with greater BMI. The rest of the descriptive data (sex, age, weight, and height) were excluded from the prediction model according to the pre-established values (P_in_ = 0.05, and P_out_ = 0.10), suggesting that these independent variables did not predict either the body weight on the left heel or the right forefoot maximum peak pressure.

## 4. Discussion

This research is the first to relate asthma to alterations in plantar pressures in a sample of adult patients. These changes in plantar pressures can be attributed to asthma, a non-communicable disease which leads to a decrease in the joint mobility of the first metatarsophalangeal joint and the ankle joint. It has previously been shown that this respiratory pathology, rather than any other confounding variables (i.e., age, weight, or height), may produce these alterations in the foot plantar pressures [27].

Furthermore, our results reflect a decrease in the percentage of body weight on the left heel in asthmatic patients, which may be related to the postural changes characteristic of asthma, such as cervical kyphosis and advanced head position [4,8].

Another alteration observed in our research is the decrease in the right forefoot maximum peak pressure. In this regard, an increase in plantar pressures in the forefoot has been associated with shortening of the posterior leg muscles in patients with cavus foot [28]. Shortening of the posterior leg musculature is frequently associated with limited dorsiflexion of the ankle joint [27], which has previously been described in patients with asthma. It would be interesting, in future research, to study the type of foot in asthmatic patients.

A maximum pressure peak in the right forefoot, as well as a lower body weight percentage on the left heel, may be correlated, due to the transfer of body weight from the hindfoot to the forefoot. This transfer of body weight may be related to a limitation of ankle dorsiflexion [27], previously described in asthmatic patients, as well as an increase in dorsal kyphosis and a forward head position [4,8], which can lead to a change in the body's center of gravity [29] and increased pronation of the foot [30].

Based on the results of our study, a multivariate predictive analysis shows a prediction of lower body weight on the left heel in asthmatic patients. Again, this may be associated with alterations in the joint mobility of the foot [27], and postural alterations in the cervical and cranial region in asthmatic patients [4,8]. The maximum peak of plantar pressure in the right forefoot appears to be predicted by BMI in both healthy and asthmatic people. A high BMI has been related to an increase in forefoot loads, in order to improve the balance index [31].

There are many studies and reviews relating foot posture to plantar pressures [32,33]. However, there is less research on the relationship between pathologies that affect the upper body region and the consequences of alterations in plantar pressures and body posture, but, as we observed in our research, this relationship exists and deserves to be investigated in order to carry out better therapeutic approaches.

Based on our results, it seems clear that asthma is a factor that generates changes in the plantar pressures of these patients compared to healthy subjects; these changes may be related to the stability of postural control, which can be altered frequently in patients with asthma [34], as well as the contractility of the diaphragm, which can also have repercussions at the postural level [35].

Regarding our initial hypothesis, patients with asthma seem to present alterations in plantar pressures with respect to healthy subjects. The subjects with asthma in this study showed less maximum pressure in the right forefoot, and less weight on the left heel. Considering that the study subjects of both groups present similar anthropometric and morphological variables, and there are no statistical differences in weight between the groups, this finding shows that weight distribution and plantar pressures occur differently in healthy and asthma groups.

This distribution could have repercussions on hyper pressure in other subjects with a higher level of asthma, or on those who are vulnerable to other risk factors and diseases that are associated with higher plantar pressures, such as diabetes [36].

Therefore, these alterations suggest that it is necessary to protocolize biomechanical studies in subjects with asthma, to show the possible alterations in plantar pressures, which have not previously been discovered until now.

Different limitations should be considered in this study. Firstly, the sampling method in this investigation was consecutive, so a randomized sampling method could be considered in the future. Secondly, the age range of the subjects in our study is between 19 and 65 years, all of them being adult patients, but it also seems necessary to study children, since asthma has a higher prevalence in the child population [37]. Despite FEV1 being an inclusion criterion for the study groups, FEV1, and other descriptive data, such as ethnicity, and foot size and shape, were not recorded and should be considered for future studies.

In future research, the study of posture and balance should be taken into account, since previous studies have established the impact of asthma on these conditions [38]. Further studies are necessary to interpret the impact of the level of asthma on alterations in plantar pressure. In addition, it seems necessary to analyze the behavior of plantar pressure in dynamics, since shortening of the musculature has been associated with asthmatic patients [10], which may generate an alteration in their gait.

## 5. Conclusions

The findings of this study show alterations in plantar pressures in asthmatic patients compared to healthy individuals. Specifically, the subjects with asthma showed less maximum pressure in the right forefoot, and less weight on the left heel, which appear to be associated with the asthma disease.

## Figures and Tables

**Table 1:** Quantitative sociodemographic and descriptive data for patients diagnosed with asthma, healthy matched-paired controls and total sample.

Quantitative Descriptive Data	Total Group (*n* = 90) Mean ± SD (Range)	Asthma (*n* = 45) Mean ± SD (Range)	Healthy (*n* = 45) Mean ± SD (Range)	*p*-Value
Age (years)	39.98 ± 11.91 (19–65)	37.55 ± 11.44 (20–65)	42.42 ± 11.96 (19–65)	0.052 *
Weight (kg)	70.31 ± 14.20 (47–120)	70.72 ± 15.20 (48–120)	69.91 ± 13.18 (47–96)	0.791 *
Height (m)	1.65 ± 0.08 (1.50–1.85)	1.67 ± 0.09 (1.53–1.85)	1.64 ± 0.08 (1.50–1.84)	0.117 †
BMI (kg/m^2^)	25.48 ± 4.52 (17.30–39.18)	25.07 ± 4.54 (18.41–39.18)	25.90 ± 4.52 (17.30–34.72)	0.327 †
Sex (male/female)	31/59	15/30	16/29	1.00 ‡

Abbreviations: BMI, body mass index. * Student´s *t*-test for independent samples was applied. † The Mann–Whitney U test was used. ‡ Fisher’s exact test was used. In all the analyses, *p* < 0.05 (with a 95% confidence interval) was considered statistically significant.

**Table 2:** Outcome measurements for patients diagnosed with asthma, healthy matched-paired controls and total sample.

Quantitative Descriptive Data	Total Group (*n* = 90) Mean ± SD (Range)	Asthma (*n* = 45) Mean ± SD (Range)	Healthy (*n* = 45) Mean ± SD (Range)	*p*-Value
Total Surface area (cm^2^)	260.17 ± 45.14 (159.00–382.00)	260.97 ± 44.18 (159.00–359.00)	259.37 ± 46.56 (163.00–382.00)	0.868 *
Left forefoot surface area (cm^2^)	68.17 ± 12.31 (37–94)	68.40 ± 12.61 (45–94)	67.95 ± 12.13 (37–89)	0.865 *
Left heel surface area (cm^2^)	62.25 ± 13.70 (34.00–102.00)	61.42 ± 14.55 (34.00-102.00)	63.08 ± 12.89 (38.00–102.00)	0.567 *
Right forefoot surface area (cm^2^)	67.02 ± 13.38 (40.00–99.00)	68.68 ± 13.36 (46.00–99.00)	65.35 ± 14.27 (40.00–93.00)	0.240 *
Right heel surface area (cm^2^)	63.27 ± 13.38 (31.00–97.00)	62.31 ± 13.98 (32.00–92.00)	64.22 ± 14.76 (31.00–97.00)	0.525 *
Body weight on the lower left limb (%)	51.15 ± 2.73 (45.00–58.00)	54.64 ± 2.85 (45.00–58.00)	51.66 ± 2.54 (46.00–57.00)	0.066 †
Body weight on the lower right limb (%)	48.95 ± 2.86 (42.00–57.00)	49.35 ± 2.85 (42.00–55.00)	48.55 ± 2.84 (43.00–57.00)	0.119 †
Body weight on the left forefoot (%)	25.40 ± 5.67 (17.00–60.00)	26.22 ± 7.05 (19.00–60.00)	24.57 ± 3.67 (17.00–32.00)	0.501 †
Body weight on the left heel (%)	26.47 ± 3.17 (18.00–35.00)	25.75 ± 3.14 (18.00–33.00)	27.20 ± 3.05 (20.00–35.00)	0.031 †
Body weight on the right forefoot (%)	23.47 ± 3.26 (15.00–32.00)	24.11 ± 2.97 (17.00–30.00)	22.84 ± 3.44 (15.00–32.00)	0.080 †
Body weight on the right heel (%)	25.58 ± 3.86 (18.00–45.00)	25.33 ± 2.85 (20.00 – 31.00)	22.84 ± 4.69 (18.00–45.00)	0.862 †
Average peak pressure (g/cm^2^)	277.75 ± 35.85 (151–366)	274.55 ± 29.65 (218.00–354.00)	280.95 ± 41.23 (215.00–366.00)	0.400 *
Left forefoot maximum peak pressure (g/cm^2^)	537.92 ± 83.35 (145.00–895.00)	533.28 ± 74.00 (378.00–684.00)	542.55 ± 92.37 (297.00–877.00)	0.601 *
Left heel maximum peak pressure (g/cm^2^)	666.45 ± 12.07 (400.00–983.00)	655.97 ± 100.01 (424.00–872.00)	676.93 ± 127.77 (400.00–983.00)	0.389 *
Right forefoot maximum peak pressure (g/cm^2^)	498.28 ± 94.64 (246.00–732.00)	478.06 ± 101.89 (246.00–712.00)	518.51 ± 83.06 (395.00–732.00)	0.042 *
Right heel maximum peak pressure (g/cm^2^)	621.62 ± 123.52 (145.00–895.00)	637.33 ± 108.55 (418.00–865.00)	605.91 ± 136.29 (145.00–895.00)	0.230 *

* Student´s *t*-test for independent samples was applied. † The Mann–Whitney U test was used. In all the analyses, *p* < 0.05 (with a 95% confidence interval) was considered statistically significant.

**Table 3:** Bivariate correlational analyses for patients diagnosed with asthma and healthy matched-paired controls.

Sociodemographic and Descriptive Data	Body Weight on the Left Heel (%)		Right Forefoot Maximum Peak Pressure (g/cm^2^)	
	Asthma	Healthy	Asthma	Healthy
Age (years)	*r*_s_ = 0.090 *p* = 0.558	*r*_s_ = −0.400 *p* = 0.006	*r* = 0.186 *p* = 0.220	*r* = 0.035 *p* = 0.820
Weight (kg)	*r*_s_ = 0.097 *p* = 0.525	*r*_s_ = 0.024 *p* = 0.876	*r* = 0.048 *p* = 0.753	*r* = 0.399 *p* = 0.007
Height (m)	*r*_s_ = 0.20 *p* = 0.176	*r*_s_ = 0.126 *p* = 0.409	*r*_s_ = −0.168 *p* = 0.270	*r*_s_ = 0.130 *P* = 0.393
BMI (kg/m2)	*r*_s_ = −0.015 *p* = 0.921	*r*_s_ = −0.076 *p* = 0.622	*r*_s_ = 0.35 *p* = 0.017	*r*_s_ = 0.417 *p* = 0.004

Pearson’s (*r*) and Spearman’s (*r*_s_) were used. In all the analyses, *p* < 0.05 (with a 95% confidence interval) was considered statistically significant.

## Data Availability

Data will be available by request.
